# A naturalistic effectiveness study of maintenance therapies for the bipolar disorders

**DOI:** 10.1111/acps.13646

**Published:** 2023-12-10

**Authors:** Michael J. Spoelma, Joanne Leidreiter, Adam Bayes, Artin Jebejian, Gordon Parker

**Affiliations:** ^1^ Discipline of Psychiatry and Mental Health, School of Clinical Medicine University of New South Wales Sydney New South Wales Australia; ^2^ Black Dog Institute Sydney New South Wales Australia; ^3^ College Street Specialists Sydney New South Wales Australia; ^4^ Gordon Private Hospital Sydney New South Wales Australia

**Keywords:** bipolar disorder, diagnosis, lamotrigine, lithium, treatment

## Abstract

**Background:**

Treatment decision‐making for individuals with bipolar disorder can be difficult. Recommendations from clinical practice guidelines can be affected by multiple methodological limitations, while pharmaco‐epidemiological data suggest great variety in prescription practices across regions. Given these inconsistencies, this study aimed to provide an alternative perspective on the effectiveness of common bipolar disorder maintenance treatments through considering naturalistic data.

**Methods:**

A total of 246 individuals with bipolar disorder (84 bipolar I [BP‐I], 162 bipolar II [BP‐II]) were recruited through clinics and/or websites. All were euthymic and had trialled at least one mood stabiliser. They completed an online survey containing questions on demographics, clinical variables, symptomatology, and the effectiveness/side effect profiles of any mood stabilisers (MSTs) or atypical antipsychotics (AAPs) that they have taken.

**Results:**

Lithium and lamotrigine were the most commonly prescribed MSTs and the most effective at mood stabilisation. Lithium and lamotrigine appeared marginally more effective for BP‐I and BP‐II respectively, however, only the latter difference was statistically significant. Furthermore, lamotrigine had the more favourable side effect profile. Amongst the AAPs, quetiapine and olanzapine were the most commonly prescribed, but they were negligibly superior to other AAPs.

**Conclusion:**

This study clearly established a preference for lamotrigine in the maintenance treatment of BP‐II. While the literature consistently emphasises the primacy of lithium in bipolar disorder treatment, its side effect profile as observed in this study remains a concern. Future research considering moderators of treatment response and concomitant medications could help to identify further nuances to consider for treatment decision‐making.

**Significant Outcomes**

- Lithium and lamotrigine were judged by patients to be the two most effective mood stabiliser medications for maintenance treatments of bipolar disorder.
- Lamotrigine appears to be preferable for those with a bipolar II disorder, largely because of its more favourable side effect profile.

**Limitations**

- The study did not account for potential moderators or mediators of treatment response.
- All data were self‐reported and were not externally validated.
- Recruitment avenues may not have been broad enough to account for geographic effects on prescription practices.

## 1 INTRODUCTION

The management of the bipolar disorders is of great clinical importance given the significant health burden these conditions pose.^1^ For maintenance treatments, pharmacotherapy with adjunctive psychotherapy is often considered to be optimal.^2^ Pharmacotherapy is generally viewed as the central therapeutic component, with mood stabilisers (MSTs) such as lithium and anticonvulsant medications (i.e., lamotrigine, valproate, and carbamazepine) prioritised, and atypical antipsychotics (AAPs) such as quetiapine, olanzapine, and aripiprazole commonly used as alternate or additional mood stabilisers or for their antimanic properties.^3^, ^4^ Antidepressants can also be used as a primary treatment, though this is generally not recommended, largely because they can cause mood switching.^5^

To identify optimal treatments for the bipolar disorders, clinical practice guidelines (CPGs) authored by professional organisations are a common reference point for clinicians as they generally argue that an evidence‐based approach underpins their development. Lithium is the most commonly recommended first‐line treatment,^6^ but recommendations can also differ according to diagnostic subtypes, as identified previously in a review of 11 CPGs.^3^ Specifically, for bipolar I disorder (BP‐I), all CPGs recommended an MST as a first‐line treatment (most commonly lithium), with AAPs being a common adjunctive medication. While for bipolar II disorder (BP‐II), the most commonly nominated medication was lamotrigine, followed by lithium and quetiapine.

However, while their evidence‐based approaches should theoretically generate consensual recommendations, the recommendations of these 11 CPGs were more distinguished by their differences than by their similarities.^3^ Such differences could reflect emphases on different nuances of bipolar disorder (e.g., severity, dominant polarity, recency of acute mania, diagnostic subtypes) or methodological nuances integral to developing the CPGs. One quality assessment study of CPGs also noted the existence of general issues relating to developmental rigour, practical applicability, conflicts of interest and involvement of necessary stakeholders, concluding that less than half of them could be recommended for use.^7^ Finally, another key issue is that CPGs generally rely on efficacy data from randomised controlled trials (RCTs) and meta‐analyses (which often rely on RCT evidence themselves^8^). While often considered the ‘gold standard’, such studies often suffer limitations in terms of external validity,^9^ such as the tendency for common inclusion/exclusion criteria to exclude those with comorbid conditions and concomitant medications.^10^ As a result, individuals recruited for these studies often differ considerably from patients seen in clinical practice.

Thus, if we accept that clinicians make treatment decisions based on their experience of what works best, there is value in also considering naturalistic (i.e., ‘real‐world’) effectiveness studies when evaluating any treatment alone or in comparison to other treatments. Pharmaco‐epidemiological data on prescription rates for medications could potentially provide an indirect measure of naturalistic efficacy, but in practice these can vary quite widely across locations. For example, in one study directly comparing treatments for those with a bipolar disorder at two university medical centres in USA and in Italy, patients at the latter site were more commonly prescribed antipsychotics (for both subtypes), valproate or antidepressants if they had a BP‐II diagnosis, and were less commonly prescribed lamotrigine if they had a BP‐I diagnosis.^11^ Another large international study identified further geographic idiosyncrasies, including lower rates of lithium usage in North America and higher rates of typical antipsychotics in Europe.^12^

In short, CPGs can be inconsistent with each other, estimates of prescription rates can be too, and CPG recommendations are often not reflected in prescription patterns.^12^ Such factors hinder our ability to draw firm conclusions on first‐choice maintenance medications for managing the bipolar disorders. Naturalistic effectiveness data, provided either by clinicians or patients themselves, can potentially be highly informative. Aside from one recent study that defined effectiveness on the basis of hospital re‐admission,^13^ such data are rarely considered. Our current study therefore aimed to evaluate the comparative effectiveness and side effect profiles of candidate maintenance medications for managing the bipolar disorders as assessed by patients' self‐reports. In so proceeding, we also sought to consider data in relation to the differing BP‐I and BP‐II sub‐types as formalised by the DSM‐5.^14^ In addition to these broad objectives, we pursued a specific hypothesis detailed previously – namely, that lithium is superior to lamotrigine for managing BP‐I, and lamotrigine is superior to lithium for managing BP‐II.^15^

## 2 MATERIALS AND METHODS

All procedures outlined below were approved by the University of New South Wales Human Research Ethics Committee (project ID HC200192).

### 2.1 Participants

Participants were recruited through three primary methods. The first involved six clinicians (which included authors J. L., A. B., A. J., and G. P.) inviting eligible patients from their own private practices in Sydney, Australia to participate in the study. The second involved online advertisements posted to recruitment webpages, newsletters, and social media accounts affiliated with the Black Dog Institute and the former School of Psychiatry at the University of New South Wales. The third method involved a direct invitation posted in a large Facebook support group consisting of (mostly American) individuals with a bipolar disorder.

Participants were required to be aged 18 or older, fluent in English, have a diagnosis of a bipolar I or II disorder, be currently euthymic, and either currently taking or had previously taken at least one of four common mood stabilisers (i.e., carbamazepine, lamotrigine, lithium, or valproate). This last inclusion criterion reflected the centrality of MSTs as maintenance treatments for the bipolar disorders.^3^ No pre‐specified target for the sample size was set due to a lack of comparable studies that might allow a power estimate. We ceased recruitment after data had been obtained for 246 participants (84 BP‐I, 162 BP‐II, see Supplementary Table 1 for a breakdown according to recruitment avenue and diagnoses), judging that if differential responses existed such a sample size would be sufficient to show trends or significant differences.

### 2.2 Study components

Participants were provided a link to an online self‐report questionnaire hosted by Qualtrics XM (Qualtrics, Provo, Utah, USA), which they could access and complete at their own convenience. The questionnaire consisted of three sections, the first two of which sought demographic and clinical information respectively. For the former, data were collected on the participant's age, sex, marital status, educational background, employment status, and ethnicity. For the latter, data were collected on their clinician's diagnosis (i.e., bipolar I or II), the ages at which subjects experienced their first depressive and first manic/hypomanic episode, their age when first diagnosed with a bipolar condition, the length of their longest and average depressive and manic/hypomanic episodes, the percentage of time before treatment spent depressed, manic/hypomanic, and euthymic, whether they had ever been hospitalised for depression and/or mania, whether they had any first‐ or second‐degree relatives with a bipolar or unipolar mood disorder, and whether they had experienced psychotic features (i.e., delusions or hallucinations) when depressed or manic.

Participants also completed a 54‐item checklist of representative manic symptoms (each scored as either present or absent). The items came from a larger item set of potential symptoms of mania/hypomania that were previously derived by an international taskforce of bipolar disorder experts.^16^ The 54 items were then identified in an exploratory factor analysis as reflecting seven major constructs underlying manic/hypomanic symptomatology: sleep, disinhibition, distractibility, grandiosity, anger/frustration, speech and spending.^17^

The third section asked questions about medication history. For all questions, participants were asked to reflect on the effectiveness and effects of each medication independently, irrespective of whether it was part of a mono‐ or poly‐therapeutic treatment program. Participants were asked first how many medications they were taking or had taken for their bipolar disorder overall, however, we were particularly interested in the four MSTs noted previously along with nine AAPs (aripiprazole, asenapine, brexpiprazole, lurasidone, olanzapine, paliperidone, quetiapine, risperidone, and ziprasidone). Participants were therefore requested to affirm which of these 13 medications they were currently taking or had previously taken, and for each, they were asked how effective they judged the medication to be in improving depression, mania/hypomania, and their bipolar disorder overall (each was measured on a continuous scale indicating the judged percentage effectiveness, ranging from 0% to 100%). Participants were also asked whether they experienced side effects, and if so, how severe they were (on a 5‐point Likert scale from “very low” to “very high”), and whether these side effects led to them ceasing the medication. Finally, participants were asked to list their current medications, and how long they had been taking each.

### 2.3 Analyses

All analyses were conducted in R (version 4.2.1), most of which examined differences between patients with BP‐I and BP‐II conditions. To this end, two sets of diagnostic strategies were employed: the bipolar subtype diagnosis provided to participants by their clinician (C1 and C2 for BP‐I and BP‐II respectively), and diagnostic classes determined only by the presence or absence of psychotic features during manic/hypomanic episodes (P1 and P2 respectively). According to DSM‐5/DSM‐5‐TR criteria and other proposed modifications,^18^ the presence of psychotic features indicates mania, and thus a BP‐I diagnosis. This can allow for a potentially parsimonious approach to differentiating BP‐I from BP‐II. However, in our sample, 50.0% of clinically diagnosed BP‐II participants (*n* = 81) reported experiencing psychotic features during their hypomanic episodes. The P1 vs P2 assignments were therefore considered to be a more flexible diagnostic allocation in highlighting the primacy of the presence/absence of psychotic features and (given the potential for clinician misdiagnosis) offered a rough proxy measure for the upper bound of the size of the BP‐I class.

Diagnostic differences in demographic and clinical features were assessed using Mann–Whitney *U* tests (for continuous variables) and either Pearson's *χ* ^2^ tests (for most categorical variables) or Fisher's exact tests (for categorical variables with at least one expected cell count less than 5). The principal analyses sought to examine differences in medication prescription rates, treatment efficacy, and side effect profiles (i.e., prevalence, severity, and cessation rates) between the MSTs and AAPs. These were reported for the overall cohort, the C1 and C2 classes, and the P1 and P2 classes where necessary (given the common use of AAPs to treat psychosis, P1 and P2 differences on AAP variables were likely to be of trivial importance). All relevant analyses were conducted using either Mann–Whitney *U* or Pearson's *χ* ^2^ tests, with Benjamini‐Hochberg adjustments for multiple comparisons.^19^ The AAPs brexpiprazole, paliperidone, and ziprasidone were excluded from all analyses due to low numbers (i.e., *n* < 10), apart from tests that considered all AAPs combined.

For medication exposure, we hypothesised that C1/P1 participants were likely to have received more medications overall, were more likely to have received lithium and AAPs, and were less likely to have received lamotrigine than C2/P2 participants. Further, in line with previously discussed research,^15^, ^20^ we hypothesised that lithium would have a superior cost–benefit profile (i.e., considering both effectiveness and side effects) than lamotrigine for C1/P1 compared to C2/P2 participants, while the reverse pattern would be true for C2/P2 participants.

## 3 RESULTS

### 3.1 Demographic and clinical features

Table 1 presents the demographic features of the sample. In brief, the median age of the sample was 40.5 years, nearly three‐quarters were women, and the proportion of those who were university‐educated, employed, had a spouse/de facto partner, or were non‐Indigenous Australians were all roughly 60%. There were no appreciable differences between the diagnostic subtypes, except for C1 patients being slightly older than C2 patients, and P1 participants being less likely than P2 participants to be employed. Differences between the diagnostic subtypes on clinical variables, as displayed in Supplementary Table 2, are largely consistent with known BP‐I and BP‐II features.

**TABLE 1:** Demographic profiles for the whole sample and each of the diagnostic subtypes (i.e., according to clinician diagnosis and the presence/absence of psychotic features).

Variable	Overall (*n* = 246)	Clinician			Psychotic features		
		BP‐I (C1; *n* = 84)	BP‐II (C2; *n* = 162)	*p* ^a^	BP‐I (P1; *n* = 154)	BP‐II (P2; *n* = 92)	*p* ^a^
Age (years), median [IQR]	40.5 [28, 51]	44 [33, 52.25]	37 [27, 51]	0.007	40.5 [28, 50]	40 [28.75, 54]	0.397
Gender, *n* (%)				0.230			0.531
Women	178 (72.4)	65 (77.4)	113 (69.8)		114 (74.0)	64 (69.6)	
Men	67 (27.2)	19 (22.6)	48 (29.6)		40 (26.0)	27 (29.3)	
Non‐binary	1 (0.4)	0 (0.0)	1 (0.6)		0 (0.0)	1 (1.1)	
Marital status, *n* (%)				0.386			0.161
Single	75 (30.5)	27 (32.1)	48 (29.6)		47 (30.5)	28 (30.4)	
In a relationship	63 (25.6)	17 (20.2)	46 (28.4)		40 (26.0)	23 (25.0)	
Married	78 (31.7)	28 (33.3)	50 (30.9)		52 (33.8)	26 (28.3)	
Separated	12 (4.9)	7 (8.3)	5 (3.1)		9 (5.8)	3 (3.3)	
Divorced	15 (6.1)	4 (4.8)	11 (6.8)		5 (3.2)	10 (10.9)	
Widowed	3 (1.2)	1 (1.2)	2 (1.2)		1 (0.6)	2 (2.2)	
Education level, *n* (%)				0.063			0.801
Secondary school not completed	15 (6.1)	6 (7.1)	9 (5.6)		11 (7.1)	4 (4.3)	
Secondary school completed	33 (13.4)	8 (9.5)	25 (15.4)		21 (13.6)	12 (13.0)	
Trade course	36 (14.6)	16 (19.0)	20 (12.3)		24 (15.6)	12 (13.0)	
Undergraduate university course	93 (37.8)	24 (28.6)	69 (42.6)		58 (37.7)	35 (38.0)	
Postgraduate university course	69 (28.0)	30 (35.7)	39 (24.1)		40 (26.0)	29 (31.5)	
Employment status, *n* (%)				0.332			0.037
Student	24 (9.8)	5 (6.0)	19 (11.7)		17 (11.0)	7 (7.6)	
Employed full‐time	98 (39.8)	31 (36.9)	67 (41.4)		55 (35.7)	43 (46.7)	
Employed part‐time	43 (17.5)	14 (16.7)	29 (17.9)		27 (17.5)	16 (17.4)	
Unemployed	31 (12.6)	12 (14.3)	19 (11.7)		26 (16.9)	5 (5.4)	
Retired	15 (6.1)	5 (6.0)	10 (6.2)		6 (3.9)	9 (9.8)	
On pension or disability benefits	35 (14.2)	17 (20.2)	18 (11.1)		23 (14.9)	12 (13.0)	
Ethnicity, *n* (%)				0.068			0.061
Non‐indigenous Australian	150 (61.0)	45 (53.6)	105 (64.8)		90 (58.4)	60 (65.2)	
Australian Aboriginal/Torres Strait Islander	6 (2.4)	3 (3.6)	3 (1.9)		6 (3.9)	0 (0.0)	
New Zealander	3 (1.2)	0 (0.0)	3 (1.9)		0 (0.0)	3 (3.3)	
European	54 (22.0)	18 (21.4)	36 (22.2)		36 (23.4)	18 (19.6)	
North American	21 (8.5)	11 (13.1)	10 (6.2)		15 (9.7)	6 (6.5)	
Central/South American	2 (0.8)	2 (2.4)	0 (0.0)		2 (1.3)	0 (0.0)	
Asian	9 (3.7)	4 (4.8)	5 (3.1)		4 (2.6)	5 (5.4)	
African	1 (0.4)	1 (1.2)	0 (0.0)		1 (0.6)	0 (0.0)	

*Note*: C1 = clinician‐diagnosed BP‐I; C2 = clinician diagnosed BP‐II; P1 = psychotic features present during mood elevation; P2 = psychotic features not present during mood elevation.

^a^ Statistical tests: age = Mann–Whitney *U*, gender/education/employment = Pearson's *χ* ^2^, marital status/ethnicity = Fisher's exact.

### 3.2 Prescription rates

Table 2 reports the lifetime and current prescription rates of MSTs and AAPs. Of the four MSTs, lithium and lamotrigine were the most commonly prescribed. Just over 70% of all participants had received an AAP, of which quetiapine was the most frequent, with nearly half of the sample having received this medication. Lifetime exposure to quetiapine was significantly higher than for the second most received AAP, olanzapine (*χ* ^2^ = 14.9, *p* < 0.001), which in turn was higher than for the third most received, aripiprazole (*χ* ^2^ = 5.9, *p* = 0.015). Aside from lamotrigine, rates of attrition appeared quite high. However, the differences in attrition rates for both lithium (C1 33.3%, P1 36.6% vs. C2 50.0%, P2 54.5%; *χ* ^2^ = 4.12/4.03, *p* = 0.042/0.045) and the AAPs (C1 32.7% vs. C2 52.9%; *χ* ^2^ = 6.79, *p* = 0.009) were significantly higher for BP‐II patients.

**TABLE 2:** Lifetime and current prescription rates of each mood stabiliser and atypical antipsychotic for the whole sample and for each diagnostic subtype.

Medication	Whole sample (*n* = 246)		Clinician diagnosis				Psychotic features			
			Lifetime *n* (%)		Current *n* (%)		Lifetime *n* (%)		Current *n* (%)	
	Lifetime *n* (%)	Current *n* (%)	C1 (*n* = 84)	C2 (*n* = 162)	C1 (*n* = 84)	C2 (*n* = 162)	P1 (*n* = 154)	P2 (*n* = 92)	P1 (*n* = 154)	P2 (*n* = 92)
*Mood stabilisers*										
Carbamazepine	26 (10.6)	6 (2.4)	11 (13.1)	15 (9.3)	3 (3.6)	3 (1.9)	19 (12.3)	7 (7.6)	5 (3.3)	1 (1.1)
Lamotrigine	166 (67.5)	123 (50.0)	37 (44.1)	129 (79.6)	25 (29.8)	98 (60.5)	96 (62.3)	70 (76.1)	67 (43.5)	56 (60.9)
Lithium	145 (59.0)	84 (34.2)	69 (82.1)	76 (46.9)	46 (54.8)	38 (23.5)	101 (65.6)	44 (47.8)	64 (41.6)	20 (21.7)
Valproate	94 (38.2)	27 (11.0)	46 (54.8)	48 (29.6)	15 (17.9)	12 (7.4)	69 (44.8)	25 (27.2)	18 (11.7)	9 (9.8)
*Atypical antipsychotics*										
Any AAP	174 (70.7)	96 (39.0)	70 (83.3)	104 (64.2)	47 (56.0)	49 (30.3)	122 (79.2)	52 (56.5)	71 (46.1)	25 (27.2)
Aripiprazole	55 (22.4)	14 (5.7)	26 (31.0)	29 (17.9)	8 (9.5)	6 (3.7)	41 (26.6)	14 (15.2)	10 (6.5)	4 (4.4)
Asenapine	17 (6.9)	6 (2.4)	9 (10.7)	8 (4.9)	3 (3.6)	3 (1.9)	13 (8.4)	4 (4.4)	4 (2.6)	2 (2.2)
Brexpiprazole	5 (2.0)	0 (0.0)	0 (0.0)	5 (3.1)	0 (0.0)	0 (0.0)	4 (2.6)	1 (1.1)	0 (0.0)	0 (0.0)
Lurasidone	29 (11.8)	13 (5.3)	9 (10.7)	20 (12.4)	6 (7.1)	7 (4.3)	21 (13.6)	8 (8.7)	10 (6.5)	3 (3.3)
Olanzapine	79 (32.1)	15 (6.1)	47 (56.0)	32 (19.8)	11 (13.1)	4 (2.5)	62 (40.3)	17 (18.5)	13 (8.4)	2 (2.2)
Paliperidone	8 (3.3)	0 (0.0)	5 (6.0)	3 (1.9)	0 (0.0)	0 (0.0)	6 (3.9)	2 (2.2)	0 (0.0)	0 (0.0)
Quetiapine	121 (49.2)	50 (20.3)	45 (53.6)	76 (46.9)	21 (25.0)	29 (17.9)	83 (53.9)	38 (41.3)	35 (22.7)	15 (16.3)
Risperidone	41 (16.7)	4 (1.6)	23 (27.4)	18 (11.1)	2 (2.4)	2 (1.2)	34 (22.1)	7 (7.6)	4 (2.6)	0 (0.0)
Ziprasidone	8 (3.3)	0 (0.0)	4 (4.8)	4 (2.5)	0 (0.0)	0 (0.0)	7 (4.6)	1 (1.1)	0 (0.0)	0 (0.0)

*Note*: C1 = clinician‐diagnosed BP‐I; C2 = clinician diagnosed BP‐II; P1 = psychotic features present during mood elevation; P2 = psychotic features not present during mood elevation.

The median number of medications prescribed overall for C1/P1 patients was six (C1 IQR = 4–10; P1 IQR = 4–9.5), compared to five (IQR = 3–7) and four (IQR = 2.5–6) for C2 and P2 patients respectively. C1 and P1 participants were also prescribed significantly more medications than their respective counterparts (Mann–Whitney *p*'s = 0.012, 0.0002). Similar patterns emerged when considering our 13 medications of interest, with C2/P2 patients (median = 3, IQR's = 2–4) prescribed significantly less of these medications than C1/P1 patients (C1 median = 4, IQR = 2.25–6; P1 median = 3.5, IQR = 2–5) (Mann–Whitney *p*'s = 0.00007, 0.0004). More specific analyses for diagnostic differences in the prescription rates for lithium, lamotrigine, and AAPs are displayed in Table 3. Lithium and AAPs were prescribed more often for C1/P1 compared to C2/P2 patients, while the opposite relationship was shown for lamotrigine. These effects were particularly pronounced in relation to clinician diagnoses, with the odds of C1 patients being prescribed lithium and C2 patients being prescribed lamotrigine being roughly five times higher than those with their respective alternative diagnostic subtype.

**TABLE 3:** Results of tests for diagnostic differences in prescription rates of lithium, lamotrigine, and all atypical antipsychotics.

Groups	Medication	% Taken		Overall test		Effect size	
		BP‐I	BP‐II	*χ* ^2^	*p*	OR	95% CI
C1 vs. C2	AAPs	83.3	64.2	9.8	0.0053	2.8	(1.40, 5.82)
	Lamotrigine	44.0	79.6	31.9	<0.0001	4.9	(2.68, 9.20)
	Lithium	82.1	46.9	28.4	<0.0001	5.2	(2.66, 10.58)
P1 vs. P2	AAPs	79.2	56.5	14.3	0.0006	2.9	(1.60, 5.38)
	Lamotrigine	62.3	76.1	5.0	0.0259	1.9	(1.04, 3.61)
	Lithium	65.6	47.8	7.5	0.0123	2.1	(1.19, 3.64)

*Note*: *p*‐values for each group of three tests (i.e., C1 vs. C2 and P1 vs. P2) were adjusted for multiple comparisons using the Benjamini‐Hochberg procedure. For all ORs, the reference category is the diagnosis with the lesser prevalence. C1 = clinician‐diagnosed BP‐I; C2 = clinician diagnosed BP‐II; P1 = psychotic features present during mood elevation; P2 = psychotic features not present during mood elevation.

### 3.3 Medication efficacy

#### 3.3.1 Effectiveness

For the MSTs, lithium and lamotrigine were consistently perceived to be superior to both valproate and carbamazepine, as reported in Supplementary Table 3. In relation to our specific hypothesis that lithium and lamotrigine are more effective for BP‐I and BP‐II respectively, the mean efficacy ratings displayed in Table 4 were in the expected directions but none of the analyses were significant after adjusting for multiple comparisons (*p* range 0.12–0.16). Unadjusted *p*‐values did show some evidence for the superiority of lamotrigine in those without psychotic features (*p* = 0.042), while a similar relationship for C2 patients just exceeded the threshold for statistical significance (*p* = 0.058).

**TABLE 4:** Overall efficacy for each medication for the whole sample and each diagnostic subtype.

Medication	Mean efficacy (SD)				
	Overall (*n* = 246)	C1 (*n* = 84)	C2 (*n* = 162)	P1 (*n* = 154)	P2 (*n* = 92)
*Mood stabilisers*					
Carbamazepine	35.5 (30.3)	38.1 (33.7)	33.7 (28.7)	37.26 (33.6)	30.86 (20.3)
Lamotrigine	58.4 (29.2)	53.3 (32.9)	59.9 (28.0)	56.23 (28.4)	61.46 (30.0)
Lithium	55.6 (32.8)	60.1 (33.2)	51.5 (32.1)	58.57 (31.3)	48.75 (35.4)
Valproate	41.5 (29.5)	45.0 (30.0)	38.2 (29.0)	41.49 (30.0)	41.60 (28.9)
*Atypical antipsychotics*					
Aripiprazole	38.3 (27.8)	41.3 (25.7)	35.7 (29.7)	40.39 (29.3)	32.21 (22.8)
Asenapine	41.4 (33.4)	42.1 (35.4)	40.5 (33.5)	41.69 (33.4)	40.25 (38.7)
Brexpiprazole	38.0 (26.2)	N/A	38.0 (26.2)	40.25 (29.7)	29.00 (N/A)
Lurasidone	52.6 (32.3)	57.2 (35.1)	50.5 (31.6)	55.24 (34.8)	45.62 (24.8)
Olanzapine	45.7 (27.0)	50.4 (27.9)	38.8 (24.4)	46.76 (27.7)	41.65 (24.6)
Paliperidone	19.6 (15.2)	19.6 (15.6)	19.7 (17.9)	22.17 (15.3)	12.00 (17.0)
Quetiapine	52.6 (28.2)	50.6 (30.4)	53.8 (27.0)	52.75 (29.2)	52.32 (26.2)
Risperidone	41.2 (34.0)	42.5 (32.9)	39.4 (36.2)	45.26 (35.3)	21.14 (17.7)
Ziprasidone	24.4 (23.8)	9.0 (8.4)	39.8 (25.0)	20.86 (23.4)	49.00 (N/A)

*Note*: C1 = clinician‐diagnosed BP‐I; C2 = clinician diagnosed BP‐II; P1 = psychotic features present during mood elevation; P2 = psychotic features not present during mood elevation.

Some clearer patterns emerged when consideration was limited to the efficacy of maintenance treatments in preventing specific mood phases (see Table 5). For depressive periods, there was clear evidence for the superiority of lamotrigine for both C2 and P2 assigned patients compared to their C1/P1 counterparts (both *p*'s = 0.002). However, a similar relationship for lithium and C1/P1 patients was not observed (both *p*'s = 0.74). For manic/hypomanic phases, lithium showed significantly greater efficacy for both C1 (*p* = 0.04) and P1 (*p* = 0.004) assigned patients. Lamotrigine also performed better for those without psychotic features (*p* = 0.04), though not for those with clinically diagnosed BP‐II (*p* = 0.42).

**TABLE 5:** Efficacy for each medication in the long‐term treatment of depression and mania/hypomania for the whole sample and each diagnostic subtype.

Medication	Depression					Mania/hypomania				
	Overall (*n* = 246)	C1 (*n* = 84)	C2 (*n* = 162)	P1 (*n* = 154)	P2 (*n* = 92)	Overall (*n* = 246)	C1 (*n* = 84)	C2 (*n* = 162)	P1 (*n* = 154)	P2 (*n* = 92)
*Mood stabilisers*										
Carbamazepine	35.3 (30.8)	39.0 (37.0)	32.6 (26.3)	36.1 (34.4)	33.1 (19.7)	34.7 (28.5)	32.5 (30.3)	36.3 (28.1)	36.2 (32.9)	30.7 (10.6)
Lamotrigine	60.9 (29.9)	57.2 (34.9)	61.9 (28.4)	58.4 (30.2)	64.2 (29.3)	60.5 (31.2)	55.1 (35.1)	62.0 (29.9)	57.8 (30.3)	64.1 (32.2)
Lithium	51.4 (32.6)	55.5 (33.6)	47.6 (31.4)	54.8 (31.8)	43.5 (33.5)	63.6 (32.2)	68.0 (31.0)	59.6 (32.9)	68.7 (29.1)	51.8 (36.0)
Valproate	37.6 (30.4)	41.8 (32.1)	33.6 (28.5)	37.9 (31.4)	36.8 (28.1)	50.6 (32.4)	54.0 (33.0)	47.4 (31.7)	52.7 (33.5)	44.9 (29.0)
*Atypical antipsychotics*										
Aripiprazole	38.2 (31.5)	35.7 (29.0)	40.5 (33.8)	39.6 (33.0)	34.3 (27.0)	43.0 (35.0)	45.0 (34.0)	41.3 (36.5)	44.1 (35.2)	39.9 (35.8)
Asenapine	39.1 (33.6)	36.4 (37.2)	42.1 (31.2)	40.3 (34.8)	35.3 (33.7)	47.8 (35.4)	53.1 (38.2)	41.9 (33.4)	48.5 (35.1)	45.5 (41.5)
Brexpiprazole	36.6 (27.7)	N/A	36.6 (27.7)	30.0 (27.1)	63.0 (N/A)	41.8 (36.7)	N/A	41.8 (36.7)	50.3 (36.3)	8.0 (N/A)
Lurasidone	52.0 (32.6)	55.0 (37.1)	50.7 (31.3)	54.8 (35.9)	44.8 (22.0)	56.9 (36.5)	65.3 (38.3)	53.1 (36.0)	59.9 (36.5)	49.0 (37.7)
Olanzapine	39.1 (27.2)	39.6 (29.4)	38.2 (24.1)	39.3 (29.0)	38.2 (20.3)	56.3 (31.3)	61.0 (29.4)	49.3 (33.0)	60.1 (30.2)	42.1 (32.2)
Paliperidone	17.5 (14.3)	17.4 (15.4)	17.7 (15.7)	19.5 (14.7)	11.5 (16.3)	27.9 (25.0)	24.8 (21.1)	33.0 (35.2)	32.3 (26.4)	14.5 (20.5)
Quetiapine	45.8 (30.8)	44.1 (32.2)	46.9 (30.1)	46.0 (32.6)	45.6 (26.7)	62.5 (29.2)	58.1 (32.0)	65.1 (27.3)	63.2 (29.5)	60.9 (28.9)
Risperidone	38.9 (32.4)	37.3 (29.4)	41.0 (36.7)	42.5 (33.6)	21.4 (19.0)	46.6 (36.3)	44.4 (34.9)	49.4 (38.9)	50.4 (36.9)	28.0 (28.5)
Ziprasidone	31.6 (31.3)	10.0 (7.2)	53.3 (31.4)	28.9 (32.7)	51.0 (N/A)	22.3 (22.8)	9.5 (9.5)	35.0 (26.2)	20.9 (24.2)	32.0 (N/A)

*Note*: C1 = clinician‐diagnosed BP‐I; C2 = clinician diagnosed BP‐II; P1 = psychotic features present during mood elevation; P2 = psychotic features not present during mood elevation.

In considering the AAPs, lurasidone, quetiapine, and olanzapine were consistently rated as the most effective ones. However, none of the differences between any of the AAPs reached statistical significance for any of the diagnostic subtypes (see Supplementary Table 4). Additionally, when all AAPs were considered together, there were no differences between C1 and C2 assigned patients in their efficacy ratings overall, or for depressive or manic/hypomanic episodes.

#### 3.3.2 Side effects

Supplementary Tables 5 and 6 report descriptive statistics for the side effect prevalence, severity, and side effect induced cessation rates for all medications. As displayed in Table 6, side effects were more severe and more common for lithium compared to lamotrigine. However, only C2/P2 patients were more likely to have ceased lithium than lamotrigine due to side effects. Further results from the pairwise comparisons of the other MSTs and AAPs are provided in Supplementary Tables 7 and 8.

**TABLE 6:** Results of tests for whole sample and diagnostic differences in side effect profiles between lamotrigine and lithium.

Side effect measures	Diagnosis	Group median/proportion		Test	
		Lamotrigine (*n* = 166)	Lithium (*n* = 145)	*χ* ^2^/U	*p*
Occurrence, %	All	47.0	77.9	31.37	<0.001
	C1	10.2	36.6	10.23	0.001
	C2	36.8	41.4	19.82	<0.001
	P1	29.5	53.8	14.73	<0.001
	P2	17.5	24.1	15.94	<0.001
Severity (0–5), median	C1	0	3	896.5	0.009
	C2	0	3	2839.5	<0.001
	P1	1	3	3376.5	<0.001
	P2	0	3	714.5	<0.001
Cessation as a result, %	All	30.8	46.9	4.77	0.048
	C1	41.2	41.5	0.0006	0.981
	C2	27.9	51.7	6.73	0.024
	P1	36.7	39.7	0.08	0.971
	P2	20.7	62.9	11.46	0.004

*Note*: *p*‐values for the prevalence, severity, and cessation analyses were adjusted for multiple comparisons using the Benjamini‐Hochberg procedure. Prevalence and cessation analyses used Pearson's *χ* ^2^ test, while the severity analyses used Mann–Whitney *U* tests. C1 = clinician‐diagnosed BP‐I, C2 = clinician diagnosed BP‐II, P1 = psychotic features present during mood elevation, P2 = psychotic features not present during mood elevation.

## 4 DISCUSSION

This study sought to collect naturalistic data on the prescription and treatment preferences of common medication treatments for the bipolar disorders, and to determine how such data might inform overall and subtype‐specific treatment recommendations. Such data have the potential to be highly informative for clinical management given the inconsistent interpretations and recommendations offered by CPGs. As noted, such CPG data are generally derived from RCTs, which have methodological (and especially sampling) limitations. Our current approach therefore focused on obtaining naturalistic effectiveness data, while also considering the impact of side effects. While we do not argue that naturalistic data are necessarily superior to RCTs, we do suggest that clinical understanding can be advanced by considering data from both sources.

A strength of this study was that reasonable sample sizes of individuals who had taken lithium and/or lamotrigine were obtained, and this allowed some hypotheses to be pursued which generated some important findings. Nevertheless, we note that there are several study limitations. First, we focused on overall characteristics of medication impact and did not consider the role that symptom profiles or other clinical variables above and beyond diagnosis had on treatment response. Second, all data and diagnoses were self‐reported and were not able to be externally validated. Third, recruitment occurred primarily in Australia and the United States, and given geographical differences in access to medications and their likely impact on prescription rates, it is unclear to what extent study findings can be generalised. Finally, there were several participants who identified as having experienced psychotic features (P1) but had not received a BP‐I diagnosis from a clinician (C1). As psychosis during elevated mood episodes necessarily confers BP‐I status, this was not expected. We could not establish whether this lack of overlap reflected clinician error, patient error, or the patients not providing information about self‐reported psychosis when clinicians were formulating their diagnosis. While we place more emphasis on the results of the C1/C2 diagnostic allocations, we note that naturalistic data necessarily preferences lived experience, and that these inconsistencies may not reflect issues with data accuracy, but rather differences in how clinicians and patients perceive psychosis. This would certainly be an interesting avenue for future research, but it is beyond the scope of this study.

Prescription rate data indicated that lithium and lamotrigine were the most commonly received MSTs, while quetiapine, olanzapine, and aripiprazole were the most commonly received AAPs. Such differential rates could reflect recommendations from CPGs, clinician observation, and/or marketing by pharmaceutical companies. It would be difficult to verify the latter explanation, but the former two are plausible given their relative consistency with both our efficacy data and frequently recommended treatments from CPGs.^21^, ^22^, ^23^ Meanwhile, when considering diagnostic subtypes, BP‐I and psychotic participants were more likely than BP‐II and non‐psychotic participants to have been prescribed lithium and any AAP, less likely to have been prescribed lamotrigine, and had also trialled more medications. The last finding may reflect the greater severity of a BP‐I or psychotic condition, its need for complementary MST and AAP medications, or its greater resistance to first line and subsequent medications.

Our results clearly indicated that, amongst the MSTs, lithium and lamotrigine were rated as more effective than both carbamazepine and valproate (admittedly only 26 participants had trialled carbamazepine; this may in itself reflect its perceived lack of efficacy by clinicians relative to other MSTs). Extending from this, one of our key hypotheses was that lithium would be the superior treatment for BP‐I and lamotrigine for BP‐II. When considering overall effectiveness alone, the differences were not statistically significant for any of the diagnostic subtypes considered in this study, although the associations were in the hypothesised direction. However, we did find evidence for the superiority of (i) lamotrigine in treating BP‐II depression and non‐psychotic mania/hypomania, and (ii) lithium for treating mania/hypomania in BP‐I patients. The greater effectiveness of lamotrigine for depression and lithium for mania is consistent with findings from CPGs.^21^, ^22^, ^23^ However, there are several other possibilities as to why diagnostic differences appeared for specific mood episodes but not for efficacy overall.

One of the most obvious, as previously mentioned, is this study's lack of accounting for the effect of moderators or mediators (aside from diagnosis) on treatment response. Controlling for these could produce clearer diagnostic differences, or alternative markers of treatment response independent of diagnosis. Data collected from this study (see Methods) as well as previous meta‐analyses of predictors^24^ could offer some possible explanations. One is that the choice of lithium or lamotrigine might be better made on the basis of predominant polarity rather than diagnostic subtype. Of course, these constructs are not mutually exclusive; for example, it is more common for the dominant polarity of BP‐II patients to be depression.^25^, ^26^ However, respecting this criterion would likely have little practical utility given the preponderance of individuals without any dominant polarity.^25^, ^26^ Finally, it could also reflect a limitation of the questions used to assess effectiveness in this study. While our questions were kept unsophisticated for purposes of simplicity, there is the possibility of differences in interpretation, specifically as to whether “overall” effectiveness necessarily emphasises whichever pole is more dominant/severe, or whether depression and mania/hypomania are given equal weight.

Participants reported that side effects were quite common, particularly for lithium and the AAPs. Lamotrigine was consistently one of the better tolerated medications, having a lower side effect prevalence rate and being the least likely medication to be ceased due to side effects. When compared to lithium specifically, lamotrigine had the superior profile for all participants when considering the presence and severity of side effects alone.

Considering these findings together, we found clear evidence for the superiority of lamotrigine for BP‐II participants. While appearing marginally more effective than lithium it had a distinctly less severe side effect profile, thus clearly establishing it as the preferred medication for treating BP‐II. For our BP‐I participants, lithium appeared marginally superior to lamotrigine in terms of effectiveness but again had a higher rate of side effects. Given the primacy of lithium in treatment recommendations for the bipolar disorders (and especially BP‐I),^6^, ^21^, ^22^, ^23^ our efficacy finding was consistent with that literature. But when considered with the side effect data, identifying a preferential treatment for BP‐I is not as clear as it appears for BP‐II. However, given the limited scope of this study, a greater emphasis on further nuances of treatment response, such as the role of moderators, polypharmacy effects, and the order in which medications are trialled, may reveal further practical realities that could argue more strongly for the primacy of lithium for BP‐I. This should be a key focus for future research.

To conclude, this study provides real world data on the effectiveness and side effect profiles of medications prescribed for managing the bipolar disorders. Our naturalistic data indicated a clear preference for lamotrigine in individuals with a BP‐II condition, further extending findings previously observed from clinical experience^15^ and trials,^20^ while also providing the first clear empirical evidence for this differential effect. For those with a BP‐I condition lithium appeared only slightly superior to lamotrigine in terms of efficacy. Ultimately, these findings could potentially be informative for clinical decision making when considered in conjunction with CPG recommendations.

## AUTHOR CONTRIBUTIONS

Michael J. Spoelma and Gordon Parker conceptualised and designed the study. Joanne Leidreiter, Adam Bayes, Artin Jebejian, and Gordon Parker recruited participants for the study. Michael J. Spoelma conducted the analyses and wrote the first draft of the manuscript. All authors contributed to drafting and revisions of the manuscript.

## CONFLICT OF INTEREST STATEMENT

The authors declare no conflicts of interest.

### PEER REVIEW

The peer review history for this article is available at https://www.webofscience.com/api/gateway/wos/peer-review/10.1111/acps.13646.

## Supporting information


**Data S1.** Supporting Information.

## Data Availability

The data associated with this study is available from the corresponding author upon reasonable request.
