# Supplementary material for: A naturalistic effectiveness study of maintenance therapies for the bipolar disorders
Source: Acta Psychiatr Scand. 2023 Dec 10;149(2):98–109. doi: 10.1111/acps.13646 (PMC10952660; doi:10.1111/acps.13646)
Supplement: Supplementary file 1 — Data S1. Supporting Information. [file ACPS-149-98-s001.docx]

**Supplementary Table 1.** Number of patients obtained from each recruitment avenue and their diagnostic allocations.

|  |  | **DIAGNOSES** | | | |
| --- | --- | --- | --- | --- | --- |
| **Recruitment** | **Total** | **C1** | **C2** | **P1** | **P2** |
| Patients of Authors | 74 | 16 | 58 | 35 | 39 |
| Online Advertisements | 145 | 53 | 96 | 101 | 48 |
| Facebook Group | 27 | 15 | 8 | 18 | 5 |

C1 = clinician-diagnosed BP-I, C2 = clinician diagnosed BP-II, P1 = psychotic features present during mood elevation, P2 = psychotic features not present during mood elevation.

**Supplementary Table 2.** Clinical variables for the whole sample and each diagnostic subtype.

|  |  |  | **CLINICIAN** | | |  | **PSYCHOTIC FEATURES** | | |
| --- | --- | --- | --- | --- | --- | --- | --- | --- | --- |
| **Variable (median [IQR] or n (%))** | **Overall (n = 246)** |  | **BP-I (C1; n = 84)** | **BP-II (C2; n = 162)** | **p*** |  | **BP-I (P1; n = 154)** | **BP-II (P2; n = 92)** | **p*** |
| Age at diagnosis (years) | 28 [22, 40] |  | 28 [21, 39.25] | 27 [22, 40] | 0.668 |  | 27 [21, 38] | 29.5 [23, 41.25] | 0.083 |
| Age of first depressive episode (years) | 16 [14, 22] |  | 17 [13.5, 25.5] | 16 [14, 20] | 0.517 |  | 16 [14, 22] | 17 [14, 22.75] | 0.349 |
| Age of first manic/hypomanic episode (years) | 20 [16.5, 26] |  | 21 [17, 28] | 20 [16, 24] | 0.497 |  | 19 [16, 24.25] | 22 [17.5, 29] | 0.015 |
| Length of longest depressive episode (days) | 90 [36.75, 235] |  | 90 [42, 180] | 90 [35, 270] | 0.607 |  | 90 [35, 225] | 95 [44, 257.5] | 0.849 |
| Length of longest manic/hypomanic episode (days) | 21 [10, 60] |  | 32.5 [14, 90] | 19 [7, 42] | <0.001 |  | 21.5 [14, 60] | 20.5 [7, 54.5] | 0.054 |
| Length of average depressive episode (days) | 28 [10, 60] |  | 28 [7, 60] | 28 [10, 60] | 0.833 |  | 25 [9.75, 60] | 28 [10, 53] | 0.790 |
| Length of average manic/hypomanic episode (days) | 7 [4, 16.25] |  | 14 [5, 30] | 6 [3, 14] | <0.001 |  | 7 [4, 21] | 7 [3, 14] | 0.035 |
| % of time spent depressed before treatment | 50 [30, 70] |  | 30 [15, 60] | 50 [40, 70] | <0.001 |  | 50 [30, 70] | 50 [33.75, 70] | 0.304 |
| % of time spent manic/hypomanic before treatment | 20 [10, 30] |  | 25 [15, 40] | 17.5 [10, 25] | <0.001 |  | 20 [15, 30] | 10 [10, 20] | <0.001 |
| % of time spent euthymic before treatment | 25 [10, 40] |  | 25 [13.75, 50] | 20 [10, 40] | 0.244 |  | 20 [10, 35] | 30 [15, 45] | 0.037 |
| Number of manic symptoms affirmed (/54) | 41 [34.75, 47] |  | 45 [37.5, 50.5] | 39 [34, 45] | <0.001 |  | 44 [38, 50] | 36 [29.75, 42] | <0.001 |
| Ever been hospitalised for depression |  |  |  |  | 0.282 |  |  |  | 0.429 |
| Yes | 123 (50.0) |  | 38 (45.2) | 85 (52.5) |  |  | 80 (51.9) | 43 (46.7) |  |
| No | 123 (50.0) |  | 46 (54.8) | 77 (47.5) |  |  | 74 (48.1) | 49 (53.3) |  |
| Ever been hospitalised for manic/hypomania |  |  |  |  | <0.001 |  |  |  | <0.001 |
| Yes | 97 (39.4) |  | 60 (71.4) | 37 (22.8) |  |  | 78 (50.6) | 19 (20.7) |  |
| No | 149 (60.6) |  | 24 (28.6) | 125 (77.2) |  |  | 76 (49.4) | 73 (79.3) |  |
| First-degree relatives with bipolar disorder |  |  |  |  | 0.822 |  |  |  | 0.687 |
| Yes | 75 (30.5) |  | 28 (33.3) | 47 (29.0) |  |  | 44 (28.6) | 31 (33.7) |  |
| No | 112 (45.5) |  | 40 (47.6) | 72 (44.4) |  |  | 69 (44.8) | 43 (46.7) |  |
| Don't know | 59 (24.0) |  | 16 (19.0) | 43 (26.5) |  |  | 41 (26.6) | 18 (19.6) |  |
| First-degree relatives with unipolar disorder |  |  |  |  | 0.007 |  |  |  | 0.703 |
| Yes | 136 (55.3) |  | 39 (46.4) | 97 (59.9) |  |  | 85 (55.2) | 51 (55.4) |  |
| No | 69 (28.0) |  | 33 (39.3) | 36 (22.2) |  |  | 45 (29.2) | 24 (26.1) |  |
| Don't know | 41 (16.7) |  | 12 (14.3) | 29 (17.9) |  |  | 24 (15.6) | 17 (18.5) |  |
| Second-degree relatives with bipolar disorder |  |  |  |  | 0.643 |  |  |  | 0.482 |
| Yes | 80 (32.5) |  | 30 (35.7) | 50 (30.9) |  |  | 48 (31.2) | 32 (34.8) |  |
| No | 53 (21.5) |  | 22 (26.2) | 31 (19.1) |  |  | 35 (22.7) | 18 (19.6) |  |
| Don't know | 113 (45.9) |  | 32 (38.1) | 81 (50.0) |  |  | 71 (46.1) | 42 (45.7) |  |
| Second-degree relatives with unipolar disorder |  |  |  |  | 0.048 |  |  |  | 0.493 |
| Yes | 132 (53.7) |  | 42 (50.0) | 90 (55.6) |  |  | 86 (55.8) | 46 (50.0) |  |
| No | 34 (13.8) |  | 17 (20.2) | 17 (10.5) |  |  | 20 (13.0) | 14 (15.2) |  |
| Don't know | 80 (32.5) |  | 25 (29.8) | 55 (34.0) |  |  | 48 (31.2) | 32 (34.8) |  |
| Has experienced delusions when manic/hypomanic |  |  |  |  | <0.001 |  |  |  |  |
| Yes | 141 (57.3) |  | 71 (84.5) | 70 (43.2) |  |  | 141 (91.6) |  |  |
| No | 105 (42.7) |  | 13 (15.5) | 92 (56.8) |  |  | 13 (8.4) |  |  |
| Has experienced hallucinations when manic/hypomanic |  |  |  |  | <0.001 |  |  |  |  |
| Yes | 100 (40.7) |  | 53 (63.1) | 47 (29.0) |  |  | 100 (64.9) |  |  |
| No | 146 (59.3) |  | 31 (36.9) | 115 (71.0) |  |  | 54 (35.1) |  |  |
| Has experienced delusions when depressed |  |  |  |  | 0.932 |  |  |  | <0.001 |
| Yes | 77 (31.3) |  | 26 (31.0) | 51 (31.5) |  |  | 63 (40.9) | 14 (15.2) |  |
| No | 169 (68.7) |  | 58 (69.0) | 111 (68.5) |  |  | 91 (59.1) | 78 (84.8) |  |
| Has experienced hallucinations when depressed |  |  |  |  | 0.202 |  |  |  | <0.001 |
| Yes | 53 (21.5) |  | 22 (26.2) | 31 (19.1) |  |  | 47 (30.5) | 6 (6.5) |  |
| No | 193 (78.5) |  | 62 (73.8) | 131 (80.9) |  |  | 107 (69.5) | 86 (93.5) |  |
| Psychotic features present |  |  |  |  | <0.001 |  |  |  |  |
| Yes (i.e., P1) | 154 (62.6) |  | 73 (86.9) | 81 (50.0) |  |  |  |  |  |
| No (i.e., P2) | 92 (37.4) |  | 11 (13.1) | 81 (50.0) |  |  |  |  |  |

* Statistical tests: all continuous = Mann-Whitney *U*, all categorical = Pearson’s χ^2^ (all tests were 2×2; “don’t know” options were ignored).

C1 = clinician-diagnosed BP-I, C2 = clinician diagnosed BP-II, P1 = psychotic features present during mood elevation, P2 = psychotic features not present during mood elevation.

**Supplementary Table 3.** Pairwise comparison tests comparing the effectiveness of each mood stabiliser.

|  | **C1 (n = 84)** | | |  | **C2 (n = 162)** | | |  | **P1 (n = 154)** | | |  | **P2 (n = 92)** | | |
| --- | --- | --- | --- | --- | --- | --- | --- | --- | --- | --- | --- | --- | --- | --- | --- |
|  | **La** | **Li** | **V** |  | **La** | **Li** | **V** |  | **La** | **Li** | **V** |  | **La** | **Li** | **V** |
| **OVERALL MOOD** |  |  |  |  |  |  |  |  |  |  |  |  |  |  |  |
| **C** | .0016 | .0083 | .5106 |  | .2953 | .1368 | .5778 |  | .0050 | .0831 | .6336 |  | .0375 | .0196 | .6406 |
| **La** |  | .7789 | .0001 |  |  | .2953 | .2953 |  |  | .1405 | .0001 |  |  | .3959 | .0055 |
| **Li** |  |  | .0009 |  |  |  | .0507 |  |  |  | .0234 |  |  |  | .0016 |
| **DEPRESSION** |  |  |  |  |  |  |  |  |  |  |  |  |  |  |  |
| **C** | .0008 | .0290 | .7257 |  | .2833 | .2833 | .8396 |  | .0013 | .1189 | .9291 |  | .0243 | .0386 | .7529 |
| **La** |  | .0194 | < .0001 |  |  | .8396 | .1012 |  |  | .0032 | < .0001 |  |  | .6290 | .0002 |
| **Li** |  |  | .0014 |  |  |  | .1012 |  |  |  | .0167 |  |  |  | .0009 |
| **MANIA/HYPOMANIA** |  |  |  |  |  |  |  |  |  |  |  |  |  |  |  |
| **C** | .0006 | .0003 | .0345 |  | .0901 | .0067 | .0894 |  | .0133 | .0245 | .2668 |  | .0159 | .0005 | .0821 |
| **La** |  | .2122 | .0216 |  |  | .0894 | .8796 |  |  | .8404 | .0133 |  |  | .0043 | .3392 |
| **Li** |  |  | .0028 |  |  |  | .0699 |  |  |  | .0343 |  |  |  | .0042 |

C = carbamazepine, C1 = clinician-diagnosed BP-I, C2 = clinician diagnosed BP-II, La = lamotrigine, Li = lithium, P1 = psychotic features present during mood elevation, P2 = psychotic features not present during mood elevation, V = valproate.

All pairwise comparisons were Mann-Whitney *U* tests, with each group of six tests (e.g., Overall Mood C1, Depression P2, etc.) adjusted for multiple comparisons using the Benjamini-Hochberg procedure. All statistically significant results are highlighted in yellow. Refer to Tables 4 and 5 to ascertain the means for each group in the comparisons.

**Supplementary Table 4.** Pairwise comparison tests comparing the effectiveness of each atypical antipsychotic.

|  | **WHOLE SAMPLE (n = 246)** | | | | |  | **C1 (n = 84)** | | | | |  | **C2 (n = 162)** | | | | |
| --- | --- | --- | --- | --- | --- | --- | --- | --- | --- | --- | --- | --- | --- | --- | --- | --- | --- |
|  | **As** | **L** | **O** | **Q** | **R** |  | **As** | **L** | **O** | **Q** | **R** |  | **As** | **L** | **O** | **Q** | **R** |
| **OVERALL MOOD** |  |  |  |  |  |  |  |  |  |  |  |  |  |  |  |  |  |
| **Ar** | .8939 | .2122 | .3785 | .0256 | .8939 |  | .9165 | .8027 | .8027 | .8027 | .9165 |  | .9389 | .3880 | .8056 | .0510 | .9389 |
| **As** |  | .5136 | .7392 | .3990 | .8939 |  |  | .8982 | .8150 | .8027 | .9165 |  |  | .8056 | .9389 | .6644 | .9389 |
| **L** |  |  | .4167 | .8939 | .4167 |  |  |  | .8027 | .8897 | .8027 |  |  |  | .4418 | .9389 | .6846 |
| **O** |  |  |  | .2438 | .5136 |  |  |  |  | .9165 | .8027 |  |  |  |  | .0649 | .9389 |
| **Q** |  |  |  |  | .2122 |  |  |  |  |  | .8027 |  |  |  |  |  | .3880 |
| **DEPRESSION** |  |  |  |  |  |  |  |  |  |  |  |  |  |  |  |  |  |
| **Ar** | .9734 | .3976 | .9734 | .3976 | .9734 |  | .8567 | .8171 | .8567 | .8171 | .8567 |  | .9435 | .9435 | .9435 | .9435 | .9435 |
| **As** |  | .4688 | .9734 | .7341 | .9734 |  |  | .8171 | .8567 | .8467 | .8567 |  |  | .9435 | .9435 | .9435 | .9435 |
| **L** |  |  | .3976 | .7341 | .4000 |  |  |  | .8171 | .8467 | .8171 |  |  |  | .9435 | .9435 | .9435 |
| **O** |  |  |  | .3976 | .9734 |  |  |  |  | .8171 | .8567 |  |  |  |  | .9435 | .9435 |
| **Q** |  |  |  |  | .4000 |  |  |  |  |  | .8171 |  |  |  |  |  | .9435 |
| **MANIA/HYPOMANIA** |  |  |  |  |  |  |  |  |  |  |  |  |  |  |  |  |  |
| **Ar** | .7871 | .3040 | .0947 | .0114 | .7937 |  | .7784 | .4214 | .3187 | .4214 | .9440 |  | .9298 | .6031 | .6031 | .0526 | .7695 |
| **As** |  | .5706 | .5169 | .3040 | .9454 |  |  | .7784 | .7784 | .8457 | .7784 |  |  | .6745 | .7864 | .2502 | .7864 |
| **L** |  |  | .9123 | .8796 | .5169 |  |  |  | .7784 | .7784 | .5191 |  |  |  | .9141 | .6031 | .9141 |
| **O** |  |  |  | .4400 | .3040 |  |  |  |  | .7784 | .3187 |  |  |  |  | .1841 | .9435 |
| **Q** |  |  |  |  | .0947 |  |  |  |  |  | .4214 |  |  |  |  |  | .6031 |

Ar = aripiprazole, As = asenapine, C1 = clinician-diagnosed BP-I, C2 = clinician diagnosed BP-II, L = lurasidone, O = olanzapine, Q = quetiapine.

All pairwise comparisons were Mann-Whitney *U* tests, with each group of fifteen tests (e.g., Overall C1, Depression C2, etc.) adjusted for multiple comparisons using the Benjamini-Hochberg procedure. All statistically significant results are highlighted in yellow. Refer to Tables 4 and 5 to ascertain the means for each group in the comparisons.

**Supplementary Table 5.** Side effect profiles for the whole sample (N = 246).

| **Medication** | **Prevalence**  **n (%)** | **Severity**  **mean (SD)** | **Ceased due to side effects**  **n (%)** |
| --- | --- | --- | --- |
| ***Mood Stabilisers*** |  |  |  |
| Carbamazepine | 9 (34.6) | 3.0 (1.2) | 4 (44.4) |
| Lamotrigine | 78 (47.0) | 2.9 (1.2) | 24 (30.8) |
| Lithium | 113 (77.9) | 3.4 (1.2) | 53 (46.5) |
| Valproate | 66 (70.2) | 3.4 (1.1) | 43 (64.2) |
|  |  |  |  |
| ***Atypical Antipsychotics*** |  |  |  |
| *Any AAPs* | 144 (82.8) | 3.6 (1.0) | 58 (39.7) |
| Aripiprazole | 31 (56.4) | 3.5 (1.1) | 18 (58.1) |
| Asenapine | 9 (52.9) | 3.8 (1.2) | 4 (44.4) |
| Brexpiprazole | 4 (80.0) | 3.3 (1.0) | 3 (75.0) |
| Lurasidone | 20 (69.0) | 3.1 (1.4) | 11 (55.0) |
| Olanzapine | 67 (84.8) | 4.0 (0.9) | 45 (66.2) |
| Paliperidone | 7 (87.5) | 4.0 (1.2) | 7 (87.5) |
| Quetiapine | 91 (75.2) | 3.4 (1.1) | 49 (52.7) |
| Risperidone | 33 (80.5) | 3.6 (1.1) | 22 (64.7) |
| Ziprasidone | 5 (62.5) | 4.2 (0.5) | 5 (100.0) |

Severity scores range from 1 (very low) to 5 (very high). SD = standard deviation.

**Supplementary Table 6.** Side effect profiles for each diagnostic subtype.

|  | **CLINICIAN DIAGNOSES** | | | | | | **PSYCHOTIC FEATURES** | | | | | |
| --- | --- | --- | --- | --- | --- | --- | --- | --- | --- | --- | --- | --- |
|  | **C1 (n = 84)** | | | **C2 (n = 162)** | | | **P1 (n = 154)** | | | **P2 (n = 92)** | | |
| **Medication** | **Pr**  **n (%)** | **S**  **mean (SD)** | **C**  **n (%)** | **Pr**  **n (%)** | **S**  **mean (SD)** | **C**  **n (%)** | **Pr**  **n (%)** | **S**  **mean (SD)** | **C**  **n (%)** | **Pr**  **n (%)** | **S**  **mean (SD)** | **C**  **n (%)** |
| ***Mood Stabilisers*** |  |  |  |  |  |  |  |  |  |  |  |  |
| Carbamazepine | 3 (27.3) | 3.7 (0.6) | 2 (66.7) | 6 (40.0) | 2.7 (1.4) | 2 (33.3) | 6 (31.6) | 3.5 (1.1) | 4 (66.7) | 3 (42.9) | 2.0 (1.0) | 0 (0.0) |
| Lamotrigine | 17 (46.0) | 3.5 (1.1) | 7 (41.2) | 61 (47.3) | 2.7 (1.2) | 17 (27.9) | 49 (51.0) | 3.1 (1.3) | 18 (36.7) | 29 (41.4) | 2.6 (1.0) | 6 (20.7) |
| Lithium | 53 (76.8) | 3.5 (1.2) | 22 (41.5) | 60 (79.0) | 3.3 (1.2) | 31 (50.8) | 78 (77.2) | 3.3 (1.2) | 31 (39.2) | 35 (79.6) | 3.5 (1.2) | 22 (62.9) |
| Valproate | 33 (71.7) | 3.4 (1.0) | 23 (69.7) | 33 (68.8) | 3.3 (1.1) | 20 (58.8) | 48 (69.6) | 3.3 (1.0) | 31 (63.3) | 18 (72.0) | 3.4 (1.2) | 12 (66.7) |
|  |  |  |  |  |  |  |  |  |  |  |  |  |
| ***Atypical Antipsychotics*** |  |  |  |  |  |  |  |  |  |  |  |  |
| *Any AAPs* | 59 (84.3) | 3.5 (1.0) | 17 (28.3) | 85 (81.7) | 3.6 (1.0) | 41 (47.7) | 107 (87.7) | 3.6 (1.0) | 39 (36.1) | 37 (71.2) | 3.7 (0.8) | 19 (50.0) |
| Aripiprazole | 12 (46.2) | 3.5 (1.3) | 5 (41.7) | 19 (65.5) | 3.5 (1.0) | 13 (68.4) | 23 (56.1) | 3.5 (1.1) | 13 (56.5) | 8 (57.1) | 3.5 (1.2) | 5 (62.5) |
| Asenapine | 3 (33.3) | 3.7 (1.2) | 1 (33.3) | 6 (75.0) | 3.8 (1.3) | 3 (50.0) | 6 (46.2) | 3.8 (1.3) | 3 (50.0) | 3 (75.0) | 3.7 (1.2) | 1 (33.3) |
| Brexpiprazole | 0 (N/A) | N/A | 0 (N/A) | 4 (80.0) | 3.3 (1.0) | 3 (75.0) | 3 (75.0) | 3.3 (1.2) | 2 (66.7) | 1 (100.0) | 3.0 (N/A) | 1 (100.0) |
| Lurasidone | 5 (55.6) | 2.8 (1.5) | 1 (20.0) | 15 (75.0) | 3.1 (1.5) | 10 (66.7) | 15 (71.4) | 2.9 (1.4) | 7 (46.7) | 5 (62.5) | 3.6 (1.7) | 4 (80.0) |
| Olanzapine | 43 (91.5) | 4.0 (1.0) | 28 (65.1) | 24 (75.0) | 4.0 (0.9) | 17 (68.0) | 58 (93.6) | 4.1 (0.9) | 39 (66.1) | 9 (52.9) | 3.4 (0.9) | 6 (66.7) |
| Paliperidone | 5 (100.0) | 4.4 (0.9) | 4 (80.0) | 2 (66.7) | 3.0 (1.4) | 3 (100.0) | 6 (100.0) | 4.3 (0.8) | 6 (85.7) | 1 (50.0) | 2.0 (N/A) | 1 (100.0) |
| Quetiapine | 32 (71.1) | 3.3 (1.1) | 12 (36.4) | 59 (77.6) | 3.5 (1.1) | 37 (61.7) | 67 (80.7) | 3.4 (1.2) | 33 (48.5) | 24 (63.2) | 3.5 (1.0) | 16 (64.0) |
| Risperidone | 16 (69.6) | 3.2 (1.1) | 8 (50.0) | 17 (94.4) | 4.1 (0.9) | 14 (77.8) | 26 (76.5) | 3.6 (1.1) | 16 (59.3) | 7 (100.0) | 3.9 (0.9) | 6 (85.7) |
| Ziprasidone | 1 (25.0) | 4.0 (N/A) | 1 (100.0) | 4 (100.0) | 4.3 (0.5) | 4 (100.0) | 4 (57.1) | 4.0 (0.0) | 4 (100.0) | 1 (100.0) | 5.0 (N/A) | 1 (100.0) |

C = ceased medication due to side effects, C1 = clinician-diagnosed BP-I, C2 = clinician diagnosed BP-II, P1 = psychotic features present during mood elevation, P2 = psychotic features not present during mood elevation, Pr = side effect prevalence, S = side effect severity (scores range from 1 (very low) to 5 (very high)), SD = standard deviation.

**Supplementary Table 7.** Pairwise comparison tests comparing the side effect severity of each mood stabiliser.

| **WHOLE SAMPLE (n = 246)** | | | |
| --- | --- | --- | --- |
|  | **Lamotrigine** | **Lithium** | **Valproate** |
| **Carbamazepine** | .3146 | .0001 | .0020 |
| **Lamotrigine** |  | < .0001 | < .0001 |
| **Lithium** |  |  | .3142 |
| **C1 (n = 84)** | | | |
|  | **Lamotrigine** | **Lithium** | **Valproate** |
| **Carbamazepine** | .4031 | .0323 | .0643 |
| **Lamotrigine** |  | .0323 | .0821 |
| **Lithium** |  |  | .5593 |
| **C2 (n = 162)** | | | |
|  | **Lamotrigine** | **Lithium** | **Valproate** |
| **Carbamazepine** | .5926 | .0056 | .0332 |
| **Lamotrigine** |  | < .0001 | .0018 |
| **Lithium** |  |  | .4023 |
| **P1 (n = 154)** | | | |
|  | **Lamotrigine** | **Lithium** | **Valproate** |
| **Carbamazepine** | .2766 | .0068 | .0188 |
| **Lamotrigine** |  | .0009 | .0156 |
| **Lithium** |  |  | .4841 |
| **P2 (n = 92)** | | | |
|  | **Lamotrigine** | **Lithium** | **Valproate** |
| **Carbamazepine** | .8579 | .0213 | .0731 |
| **Lamotrigine** |  | < .0001 | .0021 |
| **Lithium** |  |  | .4764 |

C1 = clinician-diagnosed BP-I, C2 = clinician diagnosed BP-II, P1 = psychotic features present during mood elevation, P2 = psychotic features not present during mood elevation.

All pairwise comparisons were Mann-Whitney *U* tests, with each group of six tests (e.g., Whole Sample, C1, etc.) adjusted for multiple comparisons using the Benjamini-Hochberg procedure. All statistically significant results are highlighted in yellow.

**Supplementary Table 8.** Pairwise comparison tests comparing the side effect severity of each atypical antipsychotic.

| **WHOLE SAMPLE (n = 246)** | | | | | |
| --- | --- | --- | --- | --- | --- |
|  | **Asenapine** | **Lurasidone** | **Olanzapine** | **Quetiapine** | **Risperidone** |
| **Aripiprazole** | 1.0000 | 0.7223 | 0.0006 | 0.1381 | 0.0555 |
| **Asenapine** |  | 0.7507 | 0.0547 | 0.3138 | 0.1939 |
| **Lurasidone** |  |  | 0.0089 | 0.3138 | 0.1553 |
| **Olanzapine** |  |  |  | 0.0053 | 0.1864 |
| **Quetiapine** |  |  |  |  | 0.3138 |
| **C1 (n = 84)** | | | | | |
|  | **Asenapine** | **Lurasidone** | **Olanzapine** | **Quetiapine** | **Risperidone** |
| **Aripiprazole** | 0.6919 | 0.9188 | 0.0008 | 0.2582 | 0.3647 |
| **Asenapine** |  | 0.6919 | 0.0058 | 0.2582 | 0.2958 |
| **Lurasidone** |  |  | 0.0077 | 0.3791 | 0.4464 |
| **Olanzapine** |  |  |  | 0.0008 | 0.0024 |
| **Quetiapine** |  |  |  |  | 0.9188 |
| **C2 (n = 162)** | | | | | |
|  | **Asenapine** | **Lurasidone** | **Olanzapine** | **Quetiapine** | **Risperidone** |
| **Aripiprazole** | 0.6680 | 0.9115 | 0.4113 | 0.6145 | 0.0631 |
| **Asenapine** |  | 0.6680 | 0.9584 | 0.9115 | 0.6145 |
| **Lurasidone** |  |  | 0.6145 | 0.6543 | 0.0785 |
| **Olanzapine** |  |  |  | 0.6543 | 0.4113 |
| **Quetiapine** |  |  |  |  | 0.0785 |

C1 = clinician-diagnosed BP-I, C2 = clinician diagnosed BP-II.

All pairwise comparisons were Mann-Whitney *U* tests, with each group of fifteen tests (e.g., Whole Sample, C1, C2) adjusted for multiple comparisons using the Benjamini-Hochberg procedure. All statistically significant results are highlighted in yellow.
